# Genome Comparisons of *Candida glabrata* Serial Clinical Isolates Reveal Patterns of Genetic Variation in Infecting Clonal Populations

**DOI:** 10.3389/fmicb.2019.00112

**Published:** 2019-02-12

**Authors:** Laia Carreté, Ewa Ksiezopolska, Emilia Gómez-Molero, Adela Angoulvant, Oliver Bader, Cécile Fairhead, Toni Gabaldón

**Affiliations:** ^1^Bioinformatics and Genomics Programme, Centre for Genomic Regulation, Barcelona Institute of Science and Technology, Barcelona, Spain; ^2^Departament de Ciències Experimentals i de la Salut, Universitat Pompeu Fabra (UPF), Barcelona, Spain; ^3^Institute for Medical Microbiology, University Medical Center Göttingen, Göttingen, Germany; ^4^Génétique Quantitative et Évolution Le Moulon, INRA, Université Paris-Sud, CNRS, AgroParisTech, Orsay, France; ^5^Parasitology and Mycology Department, Bicêtre University Hospital, Paris-Sud University, Le Kremlin-Bicêtre, France; ^6^Institució Catalana de Recerca i Estudis Avançats, Barcelona, Spain

**Keywords:** candidiasis, *Candida glabrata*, clinical isolates, resistance, genome sequencing, genome variation

## Abstract

*Candida glabrata* is an opportunistic fungal pathogen that currently ranks as the second most common cause of candidiasis. Although the mechanisms underlying virulence and drug resistance in *C. glabrata* are now starting to be elucidated, we still lack a good understanding of how this yeast adapts during the course of an infection. Outstanding questions are whether the observed genomic plasticity of *C. glabrata* plays a role during infection, or what levels of genetic variation exist within an infecting clonal population. To shed light onto the genomic variation within infecting *C. glabrata* populations, we compared the genomes of 11 pairs and one trio of serial clinical isolates, each obtained from a single patient. Our results provide a catalog of genetic variations existing within clonal infecting isolates, and reveal an enrichment of non-synonymous changes in genes encoding cell-wall proteins. Genetic variation and the presence of non-synonymous mutations and copy number variations accumulated within the host, suggest that clonal populations entail a non-negligible level of genetic variation that may reflect selection processes that occur within the human body. As we show here, these genomic changes can underlie phenotypic differences in traits that are relevant for infection.

## Introduction

Infections caused by fungal pathogens are becoming an increasingly serious medical problem. It is estimated that invasive fungal infections can kill around 1.5 million people every year (Brown et al., 2012). The incidence of opportunistic fungal pathogens has increased during the last years, partly owing to medical progress. Factors contributing to this increase include, among many others, extensive use of antibiotics, increased survival of immunocompromised patients, elevated use of invasive clinical procedures (such as the use of catheters, neonatal intensive care or organ transplantation), and the use of immunosuppressive chemotherapy (Pfaller and Diekema, 2007). *Candida* species are the most common source of hospital-acquired invasive fungal infections (Richardson and Lass-Flörl, 2008). Among pathogenic *Candida* species, the most prevalent in human infections are *Candida albicans* and *Candida glabrata*, usually in this order. A limited number of antifungal drugs – i.e., azoles, polyenes, echinocandins, and flucytosine (Robbins et al., 2017) – are available to treat infections caused by these species. However, the efficacy of these drugs is sometimes limited, mostly due to late diagnostics of the invasive fungal infection and, furthermore, resistance to drugs, which is in the rise (Robbins et al., 2017; Ksiezopolska and Gabaldón, 2018). Development of antifungal resistance during treatment is another possible cause of treatment failure, but we lack a comprehensive understanding of how this or other adaptive processes develop during the course of an infection. Similarly, there is very little information on what is the existing genetic diversity in pathogen populations infecting the same patient.

In this context, the study of co-isolates or serially sampled isolates obtained during the course of an infection can serve to trace variations at the genetic or physiological levels that are of relevance to understand the disease and treatment outcomes. Today, next generation sequencing technologies allow tackling this from the perspective of the entire genome sequence of the isolated strains. In genome studies of the other major yeast pathogen, *C. albicans*, serial isolates allowed the identification of several alterations that may have contributed to drug resistance over the treatment course, including segmental aneuploidies (Selmecki et al., 2006), loss of heterozygosity (LOH) in large parts of the chromosomes (Dunkel et al., 2008), and alterations at the gene level. However, these studies could not differentiate between mutations that occurred during the course of infection or selection over pre-existing standing variation. Despite the usually clonal nature of clinical isolates obtained from the same patient, it is known that there exist significant variation within *C. albicans* samples, suggesting that selection to become more resistant to drugs can shape the genetic variation of infecting populations (Ford et al., 2015; Hirakawa et al., 2015).

*Candida glabrata* shows remarkable differences with *C. albicans* (Gabaldón and Carreté, 2016). These include the fact that *C. glabrata* is a haploid organism and thus, with the exception of aneuploidies, LOH does not play a role. In addition, *C. glabrata* presents different infection strategies and antifungal properties as compared to *C. albicans*. The *C. glabrata* genome harbors several members of a specific gene family encoding epithelial adhesin proteins (EPA genes), which are considered a key factor in its ability to infect humans (Cormack and Falkow, 1999; Roetzer et al., 2011; Gabaldón et al., 2013; Vale-Silva et al., 2017). Another ability of *C. glabrata* that has been confirmed recently, using comparison of whole genome sequences, is that genetically diverse lineages can recombine, leading to genetic admixture (Dodgson et al., 2005; Carreté et al., 2018). In a recent study, we have shown that *C. glabrata* isolates can be ascribed to at least seven genetically distinct clades that lack a geographical structure (Carreté et al., 2018). Importantly, this study also revealed that *C. glabrata* is likely able to undergo mating and introgression from differentiated strains (Carreté et al., 2018). Although such studies show evidence for relatively recent recombination (i.e., unique to one or few isolates within a clade), it is unknown whether this recombination can occur in the course of human infection or commensalism. Several recent studies have compared genome sequences from *C. glabrata* isolates obtained from the same patient (Singh-Babak et al., 2012; Biswas et al., 2017; Håvelsrud and Gaustad, 2017; Vale-Silva et al., 2017; Barber et al., 2018; Carreté et al., 2018). Most of these analyses revealed very little genetic variation, supporting the idea that a single clonal population colonizes different body sites and jointly contributes to infection. Although each of these studies is very valuable, they all focused on different aspects and used different methodologies, which makes it difficult to assess whether there are common trends in the mutational landscape of infecting *C. glabrata* populations. If present, such common trends may reveal relevant processes in the adaptation of *C. glabrata* strains during the course of infection.

Here, we set out to search for global patterns of genetic variation between strains from the same patient, as these may reveal selective factors that act at the human host. For this, we reanalyzed previously available genomic data of eleven matched pairs of *C. glabrata* isolates, each from a different single patient. In addition, we sequenced a trio of serially isolated strains from the same patient obtained over the course of a week of acute infection. The analysis of single nucleotide polymorphisms (SNPs) revealed enrichment in fungal cell wall proteins and the presence of high levels of standing genetic variation.

## Materials and Methods

### Strains

The collection of 25 *C. glabrata* strains used for the analyses in this study are listed in Table 1. Three strains; SAT01BAL (synonym EF54001Bal), SAT02PL (EF54001Per), and SAT03BC (EF54001Blo) were sequenced in this study (see below). They correspond to isolates from bronchiolo-alveolar lavage (BAL), peritoneal fluid (PL), and blood culture (BC) samples collected from the same patient, respectively. This patient (age range 55–60) was hospitalized in 2007 in a hematology department, in Paris area (France) for treatment of an acute myoblastic leukemia. At the time of the initial sampling, he had received antifungal prophylaxis with fluconazole already for 2 weeks. The second and third samples were obtained one and 6 days, respectively, after the first sample. The samples were obtained during routine clinical practice in accordance to clinician’s prescription at the sampling time, and therefore they did not require patient’s consent for further research according to the French law at that time (Loi bioéthique 2004, article L1243-3 referring only to human biological samples). Moreover, the law provides a legal exception to the principle of written consent: where the care is re-qualified for research, a lack of adequate patient opposition is enough. Exception can be made when the patient cannot be found or has died which is the case for the SAT patient who passed away during hospitalization.

**Table 1:** Information about *C. glabrata* isolates.

Strains	Synonymous ID	Patient	Isolation day	Site	Country	ST	Additional details if any	Source data
B1012M	EB1012MouC	Patient 1	0	Oral	Belgium	65	Crohn disease	Enache-Angoulvant et al., 2010
B1012S	EB1012StoC	Patient 1	0	Stool	Belgium	65	Crohn disease	Enache-Angoulvant et al., 2010
EB101M	EB0101MouC	Patient 2	0	Oral	Belgium	19		Enache-Angoulvant et al., 2010
BO101S	EB0101StoC	Patient 2	0	Stool	Belgium	19		Enache-Angoulvant et al., 2010
CANGA1A		Patient 3	0	Blood	Norway	6		Håvelsrud and Gaustad, 2017
CANGA1B		Patient 3	90	Blood	Norway	6		Håvelsrud and Gaustad, 2017
CANGA2A		Patient 4	0	Blood	Norway	6		Håvelsrud and Gaustad, 2017
CANGA2B		Patient 4	90	Blood	Norway	6		Håvelsrud and Gaustad, 2017
CANGA3A		Patient 5	0	Blood	Norway	148		Håvelsrud and Gaustad, 2017
CANGA3B		Patient 5	90	Blood	Norway	148		Håvelsrud and Gaustad, 2017
CMRL1		Patient 6	0	Blood	Australia	16		Biswas et al., 2017
CMRL2		Patient 6	21	Blood	Australia	16		Biswas et al., 2017
CMRL3		Patient 7	0	Blood	Australia	145		Biswas et al., 2017
CMRL4		Patient 7	30	Blood	Australia	145		Biswas et al., 2017
CMRL5		Patient 8	0	Pelvis	Australia	New ST		Biswas et al., 2017
CMRL6		Patient 8	12	Urine	Australia	New ST		Biswas et al., 2017
P35_1		Patient 9	0	Oral	Taiwan	136	HIV positive	Lin et al., 2007
P35_2		Patient 9	90	Oral	Taiwan	136	HIV positive	Lin et al., 2007
SAT01BAL	EF54001Bal	Patient 10	0	Bronchiolo-alveolar lavage	France	55	Leukemia	This project
SAT02PL	EF54001Per	Patient 10	1	Peritoneal fluid	France	55	Leukemia	This project
SAT03BC	EF54001Blo	Patient 10	6	Blood	France	55	Leukemia	This project
DSY562		Patient 11	0	Oral	Switzerland	8	HIV positive, patient with oropharyngeal *C. glabrata* infection	Vale-Silva et al., 2017
DSY565		Patient 11	50	Oral	Switzerland	8	HIV positive, patient still with oropharyngeal candidiasis	Vale-Silva et al., 2017
NRZ-2016-057		Patient 12	0	Blood	Germany	3	Patient with acute myeloid leukemia	Barber et al., 2018
NRZ-2016-058		Patient 12	12	Blood	Germany	3	Patient with acute myeloid leukemia	Barber et al., 2018
CBS138	ATCC2001			Stool	Belgium	15	Reference genome	Dujon et al., 2004

*Columns indicates, in this order: strain name; patient number; isolate time during the course of infection; isolation site; country of isolation; multilocus sequence typing (ST); source data*.

### Sequencing

The genome sequences for SAT01BAL, SAT02PL, and SAT03BC strains were obtained at the Ultra-sequencing core facility of the CRG, using Illumina HiSeq 2000 sequencing machines. Paired-end libraries were prepared. DNA was fragmented by nebulization or in Covaris to a size of ∼600 bp. After shearing, the ends of the DNA fragments were blunted with T4 DNA polymerase and Klenow fragment (New England Biolabs). DNA was purified with a QIAquick PCR purification kit (Qiagen). 3^′^-adenylation was performed by incubation with dATP and 3^′^-5^′^-exo-Klenow fragment (New England Biolabs). DNA was purified using MinElute spin columns (Qiagen) and double-stranded Illumina paired-end adapters were ligated to the DNA using rapid T4 DNA ligase (New England Biolabs). After another purification step, adapter-ligated fragments were enriched, and adapters were extended by selective amplification in an 18-cycle PCR reaction using Phusion DNA polymerase (Finnzymes). Libraries were quantified and loaded into Illumina flow-cells at concentrations of 7–20 pM. Cluster generation was performed in an Illumina cluster station. Sequence runs of 2× 100 cycles were performed on the sequencing instrument. Base calling was performed using Illumina pipeline software. In multiplexed libraries, we used 4 bp internal indexes (5^′^ indexed sequences). De-convolution was performed using the CASAVA software (Illumina). Sequence data of the genomes has been deposited in short read archive (SRA) with the accession number PRJNA506893.

### SNP Calling

Reads were aligned onto the reference assembly of the CBS138 strain (Dujon et al., 2004) using BWA, with the BWA-MEM algorithm with 16 as number of threads (Li and Durbin, 2010). Hard and soft reads from all the genomes were filtered out using Samtools (Li, 2011) before variant analysis. We identified SNPs using GATK v3.6 (McKenna et al., 2010) with a haploid model, filtering out clusters of 5 variants within 20 bases and low quality variants, and using thresholds for mapping quality, read depth, and allele frequency (>40, >15, and >0.9, respectively).

### Structural Variants

We used deviation from the expected depth of coverage to detect structural variants (Boeva et al., 2011). For every *C. glabrata* strain we measured the number of deleted and duplicated genes using depth of coverage analysis from Samtools and GATK v3.6 with the DepthOfCoverage variable (Li, 2011). A gene deletion was considered if the depth of coverage of the gene was below 50% of the median coverage of the given gene. For duplications and large-scale structural variants we computed the median of depth of coverage for non-overlapping 10 Kb windows. Then, we normalized the coverage for each gene using their corresponding window. A duplication was taken into account if the median coverage of a gene was two times or higher than the median coverage of the window. Chromosomes E and C from CMRL2, and strain pairs CMRL3/4 and CMRL5/6 were not included in copy variation analyses due to the presence of aneuploidies or variable patterns of coverage in the sequencing libraries, respectively.

### Phenotypic Analyses

#### Growth Curves

Strains were recovered from our glycerol stock collection and grown for 2 days at 37°C on YPD agar. Single colonies were cultivated in 15 mL YPD broth (37°C, 200 rpm, overnight). Then, each sample was diluted to an optical density (OD) at 600 nm of 0.2 in 3 mL of YPD broth and grown for 3 h more in the same conditions (37°C, 200 rpm). Dilutions were made again to have an OD at 600 nm of 0.5 in 1 mL of YPD broth in order to have the same amount of cell in all the experiments. The samples were centrifuged for 2 min at 3000 ×*g*, washed with 1 ml of sterile water and centrifuged again for 2 min more at 3000 ×*g* for a final resuspension of the pellet in 1 ml of sterile water. At the end, 5 μL of each sample was inoculated in 95 μL of the corresponding medium in a 96-well plate format. All experiments were run in triplicate.

Six different growth conditions were tested: oxidative stress was assessed by measuring the growth of the cultures on YPD broth supplemented with 10 mM H_2_O_2_, reductive stress with 2.5 mM DTT and osmotic stress with 1 M NaCl; high temperature (41.5°C), pH = 2 and pH = 9 along with the control growth in standard YPD at 37°C. Cultures were grown in 96-well plates at 37 or 41.5°C, shaking, for 20 or 72 h depending on the growth rate in each condition, and monitored to determine the optical density at 600 nm every 10 min by a TECAN Infinite^®^ M200 microplate reader. Growthcurver v0.2.1, an R package, was used to measure growth (Sprouffske and Wagner, 2016).

#### Biofilm Formation Assay

Biofilm formation was assessed as described previously (Gómez-Molero et al., 2015). Studied isolates and controls (CBS138, moderate biofilm formation capacity; PEU-382 and PEU-427, high biofilm formation capacity) were cultured overnight in YPD medium at 37°C. The optical density was determined at 600 nm (Ultrospec 1000) and adjusted to a value of 2 using sterile NaCl 0.9%. Fifty microliters aliquots of the cell suspensions were placed into 96-well polystyrol microtiter plates (Greiner Bio-One) and incubated for 24 h at 37°C. The medium was removed and the attached biofilms washed once with 200 μL distilled water. Cells were stained for 30 min in 100 μL of 0.1% (w/v) crystal violet (CV) solution. Excess CV was removed and the biofilm carefully washed once with 200 μL distilled water. To release CV from the cells, 200 μL 1% (w/v) SDS in 50% (v/v) ethanol was added and the cellular material resuspended by pipetting. CV absorbance was quantified at 490 nm using a microtiter plate reader (MRX TC Revelation). Final data is the average of the three independent biological experiments, each one with four technical repeats.

#### Antifungal Drug Susceptibility Testing

Isolates were cultured overnight on YPD agar plates. After that, antifungal drug susceptibilities toward Fluconazole, Isavuconazole, Posaconazole, Voriconazole, Micafungin, Caspofungin, 5-Fluorocytosine, and Amphotericin B were determined according to EUCAST EDef 7.1 method (Arendrup et al., 2012). The MIC values of each SAT strains were calculated according to EUCAST guidelines^1^.

### Statistical Analyses

Multiple Correspondence Analysis (MCA) was performed using ade4 package for R to establish the main relationships between all sequenced strains and the reference (Tenenhaus and Young, 1985).

## Results

### Genetic Variation in Serial Clinical Isolates

To assess genomic variability present in serial *C. glabrata* clinical isolates we obtained 22 available datasets from whole genome shotguns corresponding to eleven different isolate pairs (Table 1), each obtained from the same patient over the course of 34 days, on average (Biswas et al., 2017; Håvelsrud and Gaustad, 2017; Vale-Silva et al., 2017; Carreté et al., 2018). In addition, we sequenced three serial isolates (SAT01BAL, SAT02PL, SAT03BL, here collectively referred to as the SAT strains) obtained from different body sites over the course of a week from the same leukemic patient suffering candidiasis (Table 1). Communication between the three isolation sites is highly likely since pleural cavity (from which SAT02PL was sampled) is adjacent from the lungs and the air tract (from which SAT01BAL was sampled). It is also a sterile and highly vascularized space from which micro-organisms spread easily to blood stream (where SAT03BL was sampled). The three strains were sequenced using Illumina paired-end technology to an average coverage ranging from 463 to 575×. This represents the first trio of fully sequenced *C. glabrata* serial isolates. Including the SAT strains, genomes from a total of 25 isolates corresponding to twelve different patients (Table 1) were analyzed in a common analytical framework to avoid analytical biases. For all strains we used a read-mapping strategy against the reference genome sequence of the strain CBS138 (Dujon et al., 2004) and then assessed genome variation in terms of SNPs, short and large structural variants, and genomic re-arrangements between each pair of isolates (see section “Materials and Methods”). To provide a global comparison of these genomes with previously sequenced isolates from around the globe (Carreté et al., 2018), we analyzed the obtained SNP patterns using Multiple Correspondence Analysis (MCA, see section “Materials and Methods”). MCA analysis provided consistent results with previously published clades, and suggested that all these new strains could be ascribed to several of the previously described clades (Carreté et al., 2018): namely, pairs B1012M/B1012S and EB101M/BO101S belong to clade I; SAT strains to clade II; DSY562/DSY565 to clade III; P35_2/P35_3, CMRL1/CMRL2, and CANGA3A/CANGA3B strains belong to clade VI; NRZ-2016-057/NRZ-2016-058 strains belong to clade VII; and CMRL3/CMRL4, CMRL5/CMRL6, CANGA1A/CANGA1B, and CANGA2A/CANGA2B strains belong to clade IV (Supplementary Figure S1). Thus, in this survey all isolates from the same patient belonged to the same genetic clade.

**FIGURE 1:**
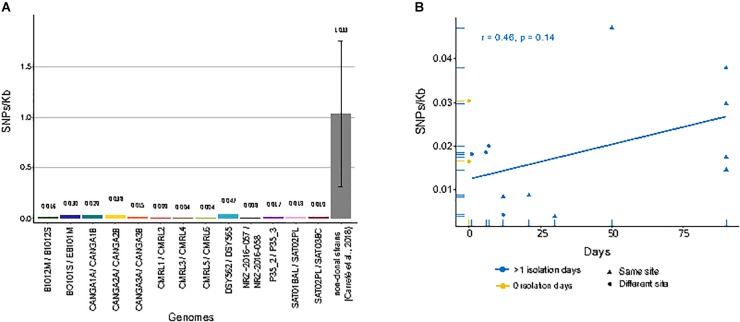
Single nucleotide polymorphisms (SNPs)/Kb between genomes of matched pairs of isolates. **(A)** Amount of different SNPs/Kb between each compared pair of genomes. Gray bar indicates the average of between all pairs of non-clonal strains from a previous study (Carreté et al., 2018). **(B)** Correlation between genome-wide mutation density (SNPs/Kb) and days span between the first isolate and second isolate in serial isolates. Yellow dots indicate pairs of strains isolated at the same day from different sites. Blue dots and triangles indicate pairs of strains isolated at different days at different sites or the same site, respectively. Blue line shows linear regression over the blue values.

When compared to the reference genome, we observed genetic distances that ranged from 2.35 SNPs/Kb in CRML6 to 6.34 SNPs/Kb in B1012M (Supplementary Table S1). We subsequently focused on detecting only those SNPs that differed between sequential isolates of the same pair or trio, as these had likely originated within the patient. Overall, we detected small differences between the two genomes of a pair, with 0.037 SNPs/Kb between CMRL3/CMRL4 as the lowest value, and 0.047 SNPs/Kb between DSY562/DSY565 as the highest value (Figure 1A). We detected short insertions and deletions (INDELs) using the same pipeline as described for SNP calling. Overall, we detect 49 private exonic INDELs that differ between pairs of isolates, which globally affected 36 genes, of which 17 encoded cell wall associated proteins (Supplementary Table S2). Intriguingly, CANGA1B, CANGA2B, and CANGA3B, share one INDEL in the same exonic position affecting gene CAGL0G04125g (named *SAG1* in *Saccharomyces cerevisiae*), involved in cell adhesion.

We next analyzed whether the number of detected SNPs correlated with the amount of time passed between isolations. A positive correlation – i.e., a larger accumulation of mutations for longer periods of time – would be expected if the serial isolates represented parent-descendant relationships. For clonal strains isolated from the same patients at different days, we observed a positive trend, but no significant correlation, between the number of private SNPs and time-span between isolations (*r* = 0.46, *p*-value = 0.14) (Figure 1B). Moreover, the genetic distance was also high between strain pairs isolated the same day but from different body sites and sometimes similar to those isolated from the same site at different days (Figure 1B). These results suggest that a significant fraction of the observed mutations, even between serially sampled isolates, were not accumulated in the time spanned between isolations. Rather they were pre-existing and represent standing genetic variation present in the infecting population. Importantly, the availability of a trio of fully sequenced serial isolates allows us, for the first time, to differentiate more specifically between the contribution of pre-existing variation and newly emerged mutations to the observed genetic differences between serially isolated strains. We observed that all private mutations in the second isolate of the series (SAT02PL) had the same alternative nucleotide in the other two strains. Assuming the three strains represent subsequent isolates of an evolving clone, this would imply a very high number of reversions, something we consider unlikely. The most parsimonious interpretation is that these SNPs represent pre-existing genetic variation within the host. Together these results suggest that the amount of genetic variation within a clonal population infecting the patient is non-negligible, and that most of the mutations found between two serial isolates may represent standing variation, rather than mutations accumulated in the time passed between the two isolations. For all the strain pairs, we asked the question on whether common trends could be observed in terms of genes that present non-synonymous SNPs between the two strains of a pair. Overall, we detected a total of 180 genes affected by non-synonymous mutations only in one of the two pair of isolates (Supplementary Table S3). Notably, we found “fungal-type cell wall” as the only enriched term among genes with non-synonymous mutations. We compared the landscape of genes with non-synonymous mutations in comparisons of matched pairs of serial isolates (184 genes) with those found when comparing independently isolated strains of the same clade (3504 genes), as described in a previous study (Carreté et al., 2018). These comparisons revealed that most (161) genes with non-synonymous mutations also present mis-sense variants in comparisons of non-clonal strains from the same clade, while only 23 were exclusive to comparisons among clonal isolates. As most of the sampled strains in the previous study are clinical isolates, these results may indicate that a subset of the identified mutations in non-clonal isolates may have accumulated during infections.

### Structural Genomic Variants

**FIGURE 2:**
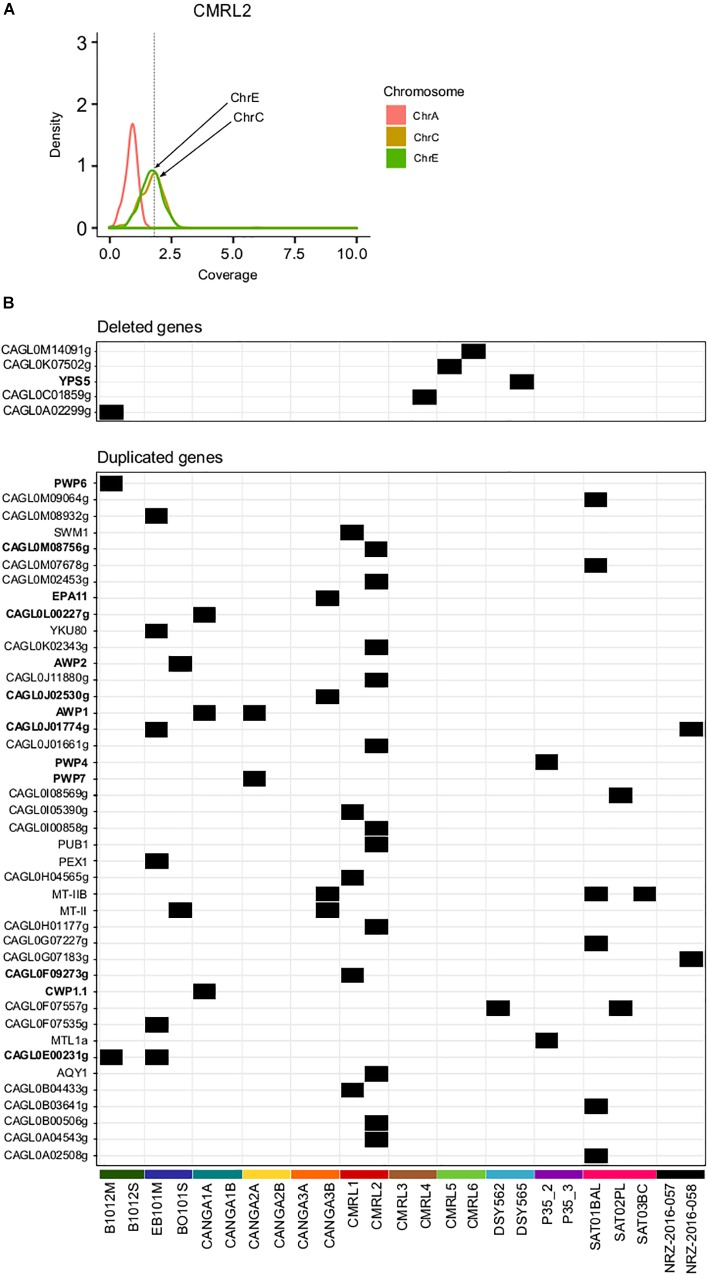
Structural genomic variation in clonal isolates. **(A)** Aneuploidy found in chromosomes C and E in CMRL2. Chromosome A as a control without any aneuploidy. *Y*-axis show the distribution of depth of coverage (density) per each chromosome. Vertical dashed line in *X*-axis indicates the threshold used to detect chromosome duplications (threshold 1.8). Chromosomes affected are marked with arrows. **(B)** Heatmap showing deleted and duplicated genes that appeared in only one of the two isolates of a pair. Gene name in bold indicates genes related to cell-wall proteins or adhesines.

Previous studies found that *C. glabrata* can undergo genomic re-arrangements as an adaptive survival mechanism (Shin et al., 2007; Muller et al., 2009; Poláková et al., 2009). We assessed large structural variants only among pairs of clonal isolates using depth of coverage analysis (see section “Materials and Methods”). This analysis revealed aneuploidies involving duplications of chromosome E and C in CMRL2 (Figure 2A). We did not detected any other large chromosomal rearrangements in the remaining pairs.

Overall, we detected 42 duplicated genes in one of the two pair of isolates (Figure 2B and Supplementary Table S4). Twelve of those duplicated genes were related with GPI-proteins and adhesins (*PWP4, PWP6, PWP7, EPA11, AWP1, AWP2*, CAGL0F09273g, *CWP1.1*, CAGL0J01774g, CAGL0J02530g, CAGL0L00227g, CAGL0M08756g). Mating type switching, as inferred from changes in coverage in mating genes was detected as previously described (Carreté et al., 2018). Previously a *MATa* to *MATalpha* switching event has been described already for the P35_2/P35_3 pair (Lin et al., 2007; Carreté et al., 2018). Here, we did not detect any other switching event in the remaining pairs.

We detected five gene deletion events between strains of a pair (Figure 2B and Supplementary Table S4). Two of the deletions appear only in the first isolate, which corroborates the previously mentioned finding that many of the differences between strains may represent standing variation, as gene deletions cannot be reversed. Among all analyzed samples, CANGA2A and CANGA2B presented deletions in the *MATalpha* cassette (CAGL0B00242g and CAGL0B01243g) and a duplication in *MATa* (CAGL0E00341g), which suggest these strains present an abnormal configuration of mating type MATa (triple **a**), as previously shown for other strains (Carreté et al., 2018). The aberrant mating type configuration of CANGA2A/CANGA2B was not reported in a previous analysis of these strains (Håvelsrud and Gaustad, 2017). In this case, as both strains have the structural variation, it is unclear whether this aberrant switching event has occurred within the patient.

### Phenotypic Variation Between Clonal Isolates and Mutations in Antifungal Response Genes

Recent studies have shown that closely related strains belonging to the same clade can have large phenotypic differences (Carreté et al., 2018). The phenotypes of three pairs of serial isolates included here, B1012M/B1012S, EB101M/BO101S, and P35_2/P35_3, had been studied previously without remarkable differences between the pairs (Carreté et al., 2018). In addition, five other pairs of isolates (DSY562/DSY565, NRZ-2016-057/NRZ-2016-058, CMRL1/CMRL2, CMRL3/CMRL4, and CMRL5/CMRL6) included in this study, have been shown to present different resistance profiles to antifungal drugs (Supplementary Table S5). DSY565, CMRL6, NRZ-2016-057, and NRZ-2016-058 were shown to be azole resistant; and CMRL2, CMRL4, and NRZ-2016-058 were shown to be echinocandin resistant (Biswas et al., 2017; Vale-Silva et al., 2017; Barber et al., 2018).

**FIGURE 3:**
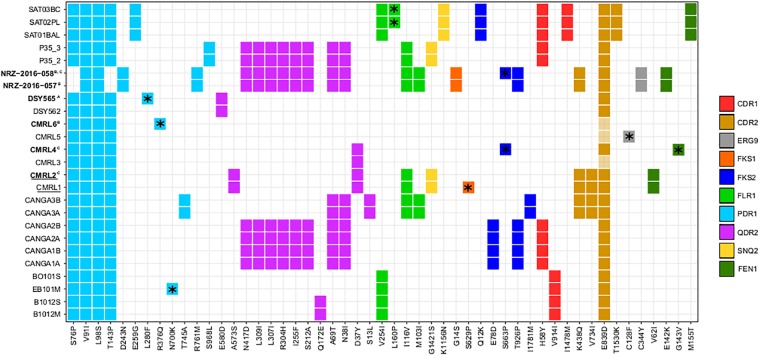
Heatmap showing non-synonymous SNPs, with respect to the reference, in genes related to drug resistance. Each column indicates mutations and each line indicates strains affected by those mutations. Different colors show distinct genes with non-synonymous mutations (CDR1, CDR2, ERG9, FKS1, FKS2, FLR1, PDR1, QDR2, SNQ2, and FEN1). *Indicates mutations that only appear in one strain of pairs. Strain names in bold indicate strains that are resistant to drugs and superscript indicates the drugs involved (A, reduced susceptibility to fluconazole; B, resistant to 5-Fluorocytosine and azoles; and C, resistant to echinocandins). Strain names that appear underlined indicate a possible mislabeling at the data deposition step from the original study (Biswas et al., 2017), see main text.

For all the strains, we analyzed non-synonymous SNPs against the reference, and focused on twelve genes which are known to affect drug resistance *(FKS1, FKS2, FEN1, SNQ2, QDR2, ERG3, ERG9, ERG11, CDR1, CDR2, FLR1*, and *PDR1*) (Sanglard et al., 2001; Tsai et al., 2006; Chen et al., 2007; Ferrari et al., 2009; Healey et al., 2012; Katiyar et al., 2012; Ni et al., 2018). We observed that *PDR1* presents the same two non-synonymous variants in all clonal strains (Figure 3). Also, with the exception of the pair NRZ-2016-057/NRZ-2016-058, the rest of the clonal strains present additionally two non-synonymous mutations (S76P and T143P). Furthermore, each of the pairs CANGA3A/CANGA3B, P35_2/P35_3, NRZ-2016-057/NRZ-2016-058 and the three SAT strains present one of five additional non-synonymous variants in *PDR1*. As shown in another study (Vale-Silva et al., 2017), the azole-resistant DSY565 presents a private non-synonymous mutation (L280F) in *PDR1*, which is known to mediate azole resistance in *C. glabrata* (Ferrari et al., 2009). The other azole resistant strain in the compared set, CMRL6, showed a different private non-synonymous mutation in *PDR1* (R376Q) and, in addition, the non-resistant CMRL5, presented a non-synonymous mutation in *ERG9* (C128F). This is consistent with the previous analysis of this strain (Biswas et al., 2017). Additionally, it is known that *FKS1* and *FKS2* mutations can confer resistance to echinocandin drugs in *C. glabrata* (Katiyar et al., 2012; Arendrup and Perlin, 2014). Consistent with previous studies (Barber et al., 2018), the second isolate of the pair NRZ-2016-057/NRZ-2016-058 presented a non-synonymous mutation in *FKS2* (S663P), which correlated with the acquisition of echinocandin resistance. The original comparison of the two echinocandin resistant strains, CMRL2 and CMRL4, with their susceptible paired strains, reported mutations in *FKS1* (S629P) and *FKS2* (S663P), respectively (Biswas et al., 2017). These coordinates belong to hot spot regions in these two genes in which mutations can confer elevated MICs (Arendrup and Perlin, 2014). We here confirmed CMRL4 *FKS2* mutation, but we could not reproduce the results of CMRL2 *FKS1* gene. Instead our analysis, confirmed by manual inspection of the mapping reads (Supplementary Figure S2), suggests that the hot spot mutation (S629P) is present in the supposedly non-resistant strain, labeled as CMRL1 (Figure 3). We suspect that mislabeling at the data deposition step may explain this incongruency. In addition, *FKS2* presented five non-private mutations (Figure 3). Given the lack of correlation of these mutations to echinocandin resistance, this suggests that these non-private mutations represent natural variation unrelated to a resistant phenotype. Mutations in the *FEN1* gene have been related to acquisition of resistance to echinocandins in *C. glabrata*, with some mutations leading to reduced susceptibility to caspofungin but not to micafungin (Healey et al., 2012). In our dataset, we observed three different non-synonymous mutations shared in three different pair of isolates: V62I in CMRL1/CMRL2, E142K in NRZ-2016-057/NRZ-2016-058, and M155T in SAT strains. Additionally, CMRL4 presents one private mutation in G143V (Figure 3). With the exception of the SAT strains, the rest of strains with non-synonymous mutations in *FEN1* presented lower susceptibility to echinocandin drugs in the second pair of isolate. This suggest that the combination of the appearance of *FKS* and *FEN1* mutations can jointly contribute to the acquisition of resistance to echinocandin drugs in *C. glabrata*.

Additionally, it has been suggested that mutations in *MSH2*, a mismatch repair gene, can influence the capacity to acquire resistance to drugs (Healey et al., 2016). In a previous analysis it was reported that DSY562 and DSY565 both carry a V239L mutation in *MSH2* and that these strains may display a faster evolutionary rate (Vale-Silva et al., 2017). We additionally found another non-synonymous mutation shared by these two strains (A942T), and four other *MSH2* non-synonymous mutations distributed in three other strain pairs (Supplementary Figure S3). Consistent with the above mentioned report, DSY562/DSY565 do seem to have an increased mutation rate (more accumulated mutations with respect to the time spanned between isolations). However, the other strains with *MSH2* mutations showed typical, or even lower, levels of divergence to their respective isolate pairs. This suggests that these mutations, at least the ones shared by both strains of a pair (N890I and L810H), do not affect the capacity for DNA repair (Supplementary Figure S3).

**FIGURE 4:**
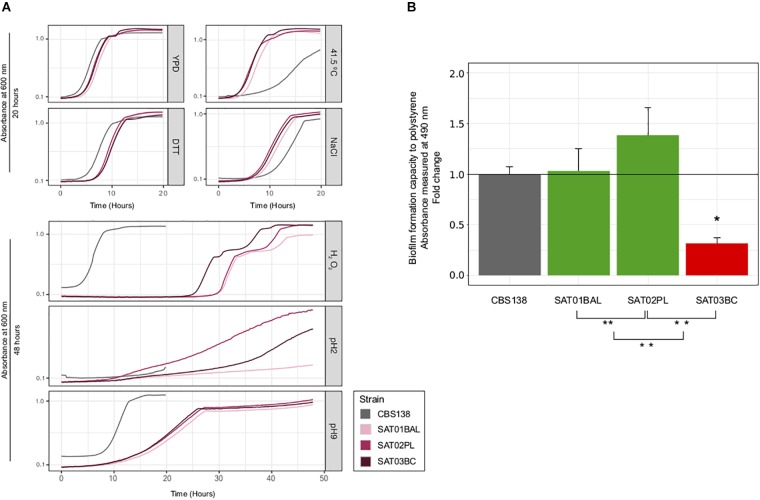
Phenotypic analysis of SAT strains. **(A)** Growth curves for seven different condition: YPD, NaCl, DTT, high temperature (41.5 T°), H_2_O_2_, basic pH (pH = 9) and acid (pH = 2). All curves were carried out at 37°C, with the exception of high temperature condition. *X*-axis shows time (in hours) and *Y*-axis shows the OD value per each condition. Strain CBS138 used as a control. At the top, growth curves that achieved the maximum carring capacity (K) before 20 h. At the bottom growth curves for conditions in which some strains presented very slow growth and the incubation time had to be extended (48 h). **(B)** Biofilm formation analysis. Barplots represent averages of three independent replicas of four technical repeats each. ^∗^ and ^∗∗^ indicates if exist significantly differences between SAT01BAL-SAT02PL, SAT02PL-SAT03BC, and SAT01BAL-SAT03BC (Student’s *t*-test, *p*-value <0.05). Statistically significant differences between reference CBS138 and SAT03BC was found (*p*-value 0.0011, red barplot).

To further assess to what extent phenotypic variation can exist within clonal populations infecting the same patient, we measured the growth of the three SAT isolates on a panel of six different stress conditions, assessed drug resistance profiles, and measured adherence. Our test for antifungal susceptibility indicated that none of the isolates had a lower susceptibility, in accordance with the lack of observed mutations in typical drug resistance genes (Figure 3 and Supplementary Table S5). Most tested conditions resulted in a similar growth of the three clonal isolates, with the exception of oxidative (hydrogen peroxide) and acidic (pH2) stress conditions (Figure 4A and Supplementary Table S6). SAT03BC and SAT02PL showed a faster growth rate as compared to the other isolates in the presence of hydrogen peroxide or low pH (pH = 2), respectively. We also analyzed differences in adherence. We observed small but significant differences between the adherence levels of SAT01BAL and SAT03BC (*p*-value 0.0011) (Figure 4B). SAT03BC was found to contain a non-synonymous mutation in *SIR4*, a protein involved in subtelomeric silencing and regulation of biofilm formation (Iraqui et al., 2004). This difference, together with the enrichment in non-synonymous SNPs related to cell wall properties (see above), may underlie the observed phenotypic variation in terms of adhesion properties, although this hypothesis will require further experimental validation.

## Discussion

To obtain an overall view of the genomic variation of *C. glabrata* in patients under treatment, we re-analyzed different samples from previous studies (Håvelsrud and Gaustad, 2017; Vale-Silva et al., 2017; Barber et al., 2018; Carreté et al., 2018). In addition we sequenced a trio of serial isolates. The availability of three clinical samples instead of two, provides us with the possibility to differentiate between newly appeared mutations between a pair of isolates and variation that likely predates the divergence of the compared strains. All serially isolated strains from the same patient belonged to the same clade as defined in previous studies (Carreté et al., 2018). Similarly, most strains within a pair showed a very low density of SNPs (0.037–0.047 SNPs/Kb), confirming their clonal nature (Biswas et al., 2017; Vale-Silva et al., 2017; Carreté et al., 2018). Aberrant mating type switching events resulting in unorthodox configurations of the mating type loci have been shown to be common among *C. glabrata* clinical isolates (Brockert et al., 2003; Carreté et al., 2018), and here we detect one additional such case. Moreover, the genetic structure of *C. glabrata* populations suggest that human may not be the natural niche for this yeast (Carreté et al., 2018). In this context an open question is whether the conditions encountered by *C. glabrata* in humans may promote switching, which often turns aberrant, as opposed to canonical switching putatively triggered by conditions more natural to *C. glabrata*. The analysis of environmental isolates could confirm or refute this idea.

Our analysis of genetic variation in serial clinical isolates indicate that despite their clonal nature, the genetic diversity within an infecting clonal population is non-negligible and of the order of hundreds of mutations between any pair of strains. In addition, our analyses of a trio of isolates reveals that the first and third isolate share mutations that are absent from the intermediate isolate. This suggests that most of the mutations found between any pair of serial isolates may represent pre-existing mutations present in the population (i.e., standing variation), rather than mutations occurred between the isolations. Finally, several non-synonymous point mutations and structural variants affect relevant genes and may potentially have phenotypic effects of relevance for the treatment and infection outcomes. With the current sampling and the lack of knowledge on the mutation rate and generation time of *C. glabrata* it is impossible to know whether this high genetic variability predated the infectious phase and represents genetic variability existing in a commensal population, or, alternatively, whether most of the observed genetic differences appeared during the course of infection. If we were to assume a per base mutation rate similar to that of haploid yeasts (estimated at 0.33 10^-9^ per base per generation) (Lynch et al., 2008), each generation one would expect 0.0041 mutations in the genome (0.33 10^-9^ × 12,3 10^6^ bases in the genome = 4.1 10^-3^). In other words 0.0000041 SNPs per Kb and generation. In our dataset we have found between 0.037 and 0.047 SNPs/Kb in pairwise comparisons of serial isolates, which would mean that these strains are separated by around 9–11.4 10^3^ generations. Further assuming a doubling time close to 1 h in *S. cerevisiae*, these would suggest that these strains are separated by 374–474 days (or that they diverged 187–237 days ago). Given the huge extrapolations and strong assumptions involved, we consider that these estimates are to be used with extreme care. Nevertheless these estimates seem to suggest that the divergence of the strains is larger than the period between isolations, reinforcing our conclusions that many of the mutations observed between any pair of serial isolates represent standing variation.

We cannot fully discard the possibility that the different serial isolates come from two independent infections from related strains. However, even if that is the case our mutation set shared by serial isolates in different patients is likely to be enriched in mutations that accumulated during infections because: (i) all isolates are taken from patients during a diagnosed fungal infection and it can be assumed that each sampled strain has been in the patient for some time; (ii) *Candida* infections are difficult to clear, particularly in immunocompromised persons, suggesting continuous infection in hospitalized patients is the most plausible scenario; (iii) genetic distances between matched serial isolates are at least one order of magnitude smaller than typical genetic distances between independent isolates belonging to the same clade; and (iv) even if recurrent infection is a possibility, a safe assumption is that most cases represent continuous infection. In such circumstances, the few cases of recurrent infection should appear as outliers, as they would be sampled from a significantly larger population. In our view, the high similarity between all the compared serial isolates makes the scenario of recurrent infection from different sources unlikely, although we cannot fully discard the possibility of a recurrent infection from the same original external reservoir, i.e., a contamination source in the hospital environment. Similarly, a large standing variation would also be compatible with the infecting strains originating from an established commensal population rather than from a recent infection with few infecting cells.

Currently, the discussion on what the natural niche of *C. glabrata* may be remains an open question (Gabaldón and Fairhead, 2018). A natural reservoir in the environment coupled with occasional commensalism in healthy humans would be compatible with reports of *C. glabrata* isolates from healthy human samples and with a large level of standing genetic variation within a patient infected by its endogenous *C. glabrata* population (Gabaldón and Fairhead, 2018). Alternatively, genetic variation could be generated during weeks of infection if the patient sustains large *C. glabrata* populations and if stress conditions promote high mutation rates and strong diversifying selection at different body sites. We found that non-synonymous mutations in genes potentially related to drug resistance were common, sometimes, but not always, correlating with experimentally determined lower susceptibilities to drugs. The phenotypic analyses for the trio of isolates performed here, revealed small but significant differences in adherence properties, and suggest possible links with observed mutations. In addition, the two invasive strains isolated from host fluids – SAT02PL from peritoneal liquid, and SAT03BC from blood – present better growth in stress conditions (low pH and oxidative stress) that could be potentially related to higher survival rate in the phagolysosome of human macrophages (Kasper et al., 2014). Another interesting finding is the lower adherence of the blood isolate (SAT03BC) as compared to the other two strains. This strain presents a non-synonymous mutation in *SIR4*, a gene involved in subtelomeric silencing and regulation of biofilm formation. A recent study (Leiva-Peláez et al., 2018) has found that *SIR3* and *SIR4* from several clinical isolates present a larger number of non-synonymous polymorphisms as compared to genes encoding other silencing proteins, and that some such polymorphisms relate to different levels of silencing of target genes. A possible scenario is thus that the observed mutation induces changes in the expressed adhesin genes, leading to lower adherence. As SAT03BC was isolated from blood it is tempting to speculate that a reduced adhesion phenotype may have enhanced the dissemination ability of this strain, thereby facilitating dispersion into the bloodstream. Nevertheless, the limited set of strains makes it difficult to find clear correlations between genetic variations and phenotypes, and all suggested relationships would require further validation.

Overall, the analysis of private SNPs across all clonal pairs shows an enrichment of non-synonymous mutations affecting cell-wall proteins. This functional enrichment was also detected among non-synonymous SNPs found in an overall comparison of clinical *C. glabrata* strains (Carreté et al., 2018). Our results, restricted to non-synonymous SNPs potentially accumulated within the host, suggest that the overall pattern present across clinical strains may result from selection processes that occur within the human body. Alternatively, genes encoding cell-wall proteins may be more mutagenic, given their subtelomeric localization and the presence of repeats. Admittedly, two or three isolates per patient represent a very poor sampling of the infecting population and our results should be confirmed with larger datasets. In this regard, proper deposition of the sequences as well as of relevant clinical data (i.e., underlying disease or treatment regime of the patient) will be key for future meta-analyses that analyze data from serial isolates from diverse studies. In our own experience, it has not always been possible to retrieve relevant information from published studies. Future studies including additional serial isolates from patients and its comparison with patterns observed in environmental isolates will undoubtedly help us to clarify how genomic variation and selection processes affect disease and treatment outcome.

## Author Contributions

EK and EG-M performed the experiments. LC performed the bioinformatics analyses. AA, OB, CF, and TG designed and supervised the different parts of the study. TG and LC wrote the first draft of the manuscript. All authors contributed to revisions of the manuscript. TG coordinated the project.

## Conflict of Interest Statement

The authors declare that the research was conducted in the absence of any commercial or financial relationships that could be construed as a potential conflict of interest.

## Footnotes

1 http://www.eucast.org/fileadmin/src/media/PDFs/EUCAST_files/AFST/Clinical_breakpoints/Antifungal_breakpoints_v_9.0_180212.pdf, accessed November 16th, 2016.

## Supplementary Material

FIGURE S1 Distribution of strains based on SNPs using a Multiple Correspondence Analysis (MCA). Each pair of strains used marked in different colors and names in plot. White circles in plot indicates strains used to provide a global comparison (Carreté et al., 2018).

FIGURE S2 Detail of read mapping on the *FKS1* gene region, visualized using IGV software (Thorvaldsdóttir et al., 2013) in the strains CMRL1, CMRL2, CMRL3, CMRL4, CMRL5, and CMRL6. Zoom-in square indicates private non-synonymous SNP S629P present only in CMRL1 (in blue).

FIGURE S3 Correlation between genome-wide mutation density (SNPs/Kb) and days spanned between the first isolate and second isolate in serial isolates. As Figure 1B but strains are colored according to the presence of non-synonymous mutations in *MSH2*.

TABLE S1 Single nucleotide polymorphisms (SNPs)/Kb value compared to reference genome CBS138. First column indicates list of *C. glabrata* isolates analyzed in this study. Second column indicates SNPs/Kb compared to reference genome (CBS138).

TABLE S2 List of genes affected by private exonic INDELs. Columns indicates, in this order: pair of strains; INDEL position; systematic name; standard name; type of mutation; description of the gene affected.

TABLE S3 List of genes affected by private non-synonymous mutations in clonal strains. Columns indicates: strain name; Systematic name of the gene affected by a private non-synonymous mutation; standard name of that gene; amino acid (AA) altered; description of the gene.

TABLE S4 List of genes deleted and duplicated in one of the two pair of isolates. First column indicates gene name; second column indicates description of the gene affected.

TABLE S5 MIC value (μg/ml) for the different strains analyzed. Columns indicates: name of the strains analyzed, isolation site, drugs tested (fluconazole, Voriconazole, Amphotericin B, Anidulafungin, Micafungin, Caspofungin, 5-fluorocystosine, Posaconazole, Itraconazole, Isavuconazole); method used; and source data.

TABLE S6 Values from phenotypic analysis. Columns indicate: sample; condition used for the phenotypic analysis; carrying capacity (k), population size at time 0 (time 0); growth rate (r); time when the population density reaches ½ K (½ K time); fastest possible generation time; area under the logistic curve measured by taking the integral logistic equation (auc_l); empirical area under the curve (auc_e); measure of the goodness of fit of the parameters for the logistic equation (sigma). See Figure 4 for more details.
