# Supplementary figures and images for: Genome Comparisons of Candida glabrata Serial Clinical Isolates Reveal Patterns of Genetic Variation in Infecting Clonal Populations
Source: Front Microbiol. 2019 Feb 12;10:112. doi: 10.3389/fmicb.2019.00112 (PMC6379656; doi:10.3389/fmicb.2019.00112)

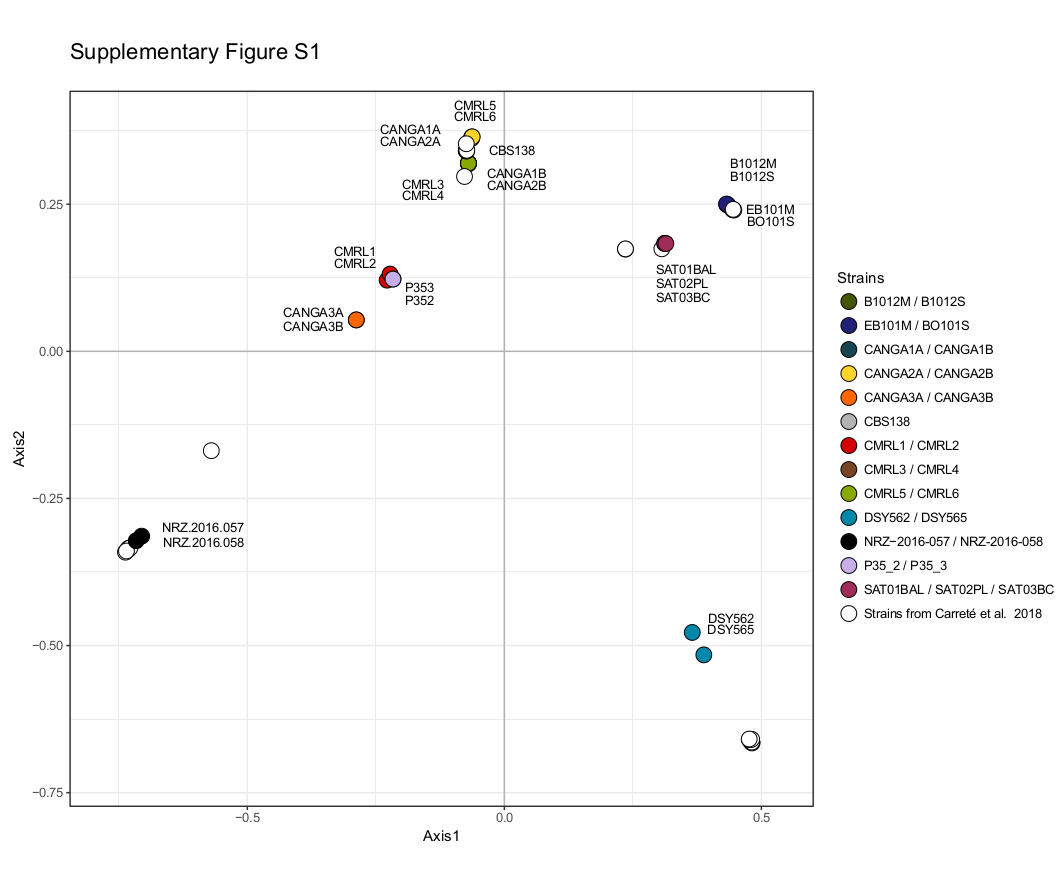

Supplement: FIGURE S1 — Distribution of strains based on SNPs using a Multiple Correspondence Analysis (MCA). Each pair of strains used marked in different colors and names in plot. White circles in plot indicates strains used to provide a global comparison (Carreté et al., 2018). [file Image_1.TIFF]

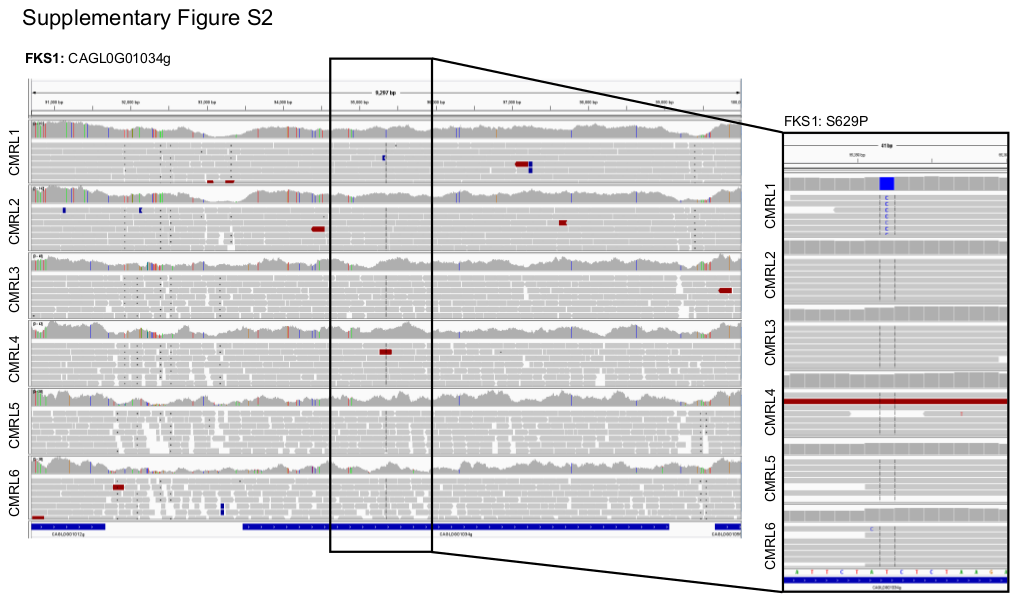

Supplement: FIGURE S2 — Detail of read mapping on the FKS1 gene region, visualized using IGV software (Thorvaldsdóttir et al., 2013) in the strains CMRL1, CMRL2, CMRL3, CMRL4, CMRL5, and CMRL6. Zoom-in square indicates private non-synonymous SNP S629P present only in CMRL1 (in blue). [file Image_2.TIFF]

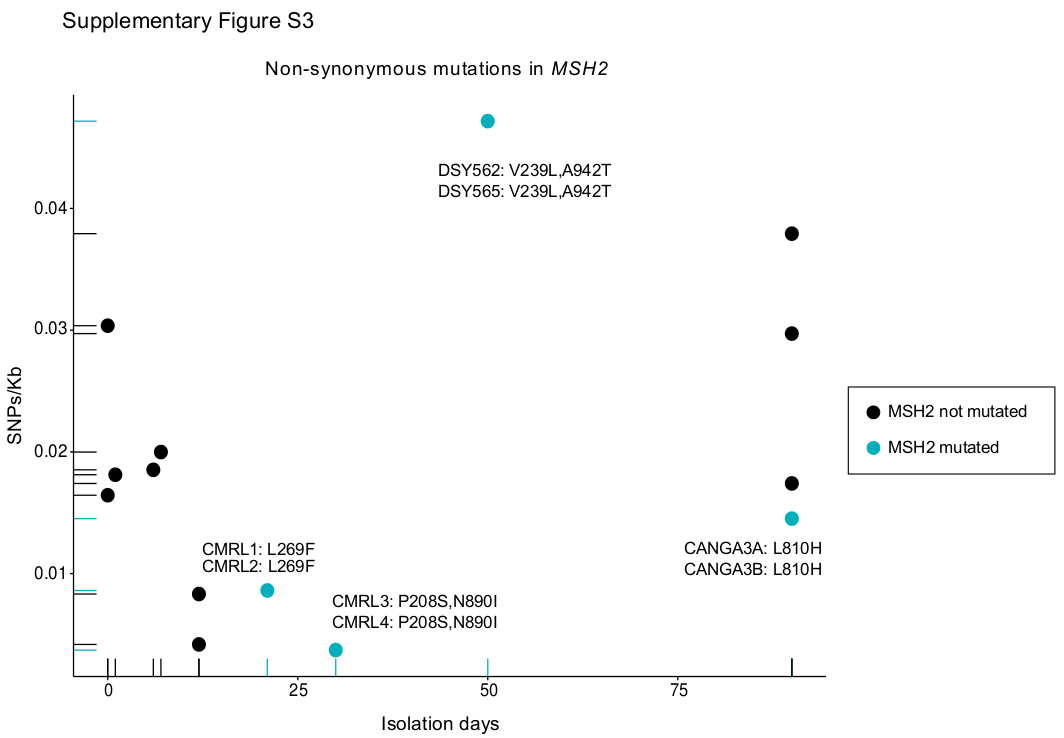

Supplement: FIGURE S3 — Correlation between genome-wide mutation density (SNPs/Kb) and days spanned between the first isolate and second isolate in serial isolates. As Figure 1B but strains are colored according to the presence of non-synonymous mutations in MSH2. [file Image_3.TIFF]
